# Supplementary material for: Transgenerational Plasticity Enhances the Tolerance of Duckweed (Lemna minor) to Stress from Exudates of Microcystis aeruginosa
Source: Int J Mol Sci. 2024 Dec 4;25(23):13027. doi: 10.3390/ijms252313027 (PMC11641799; doi:10.3390/ijms252313027)
Supplement: Supplementary file 1 [file ijms-25-13027-s001.zip › Supplementary Figures.pptx]

## Slide 1
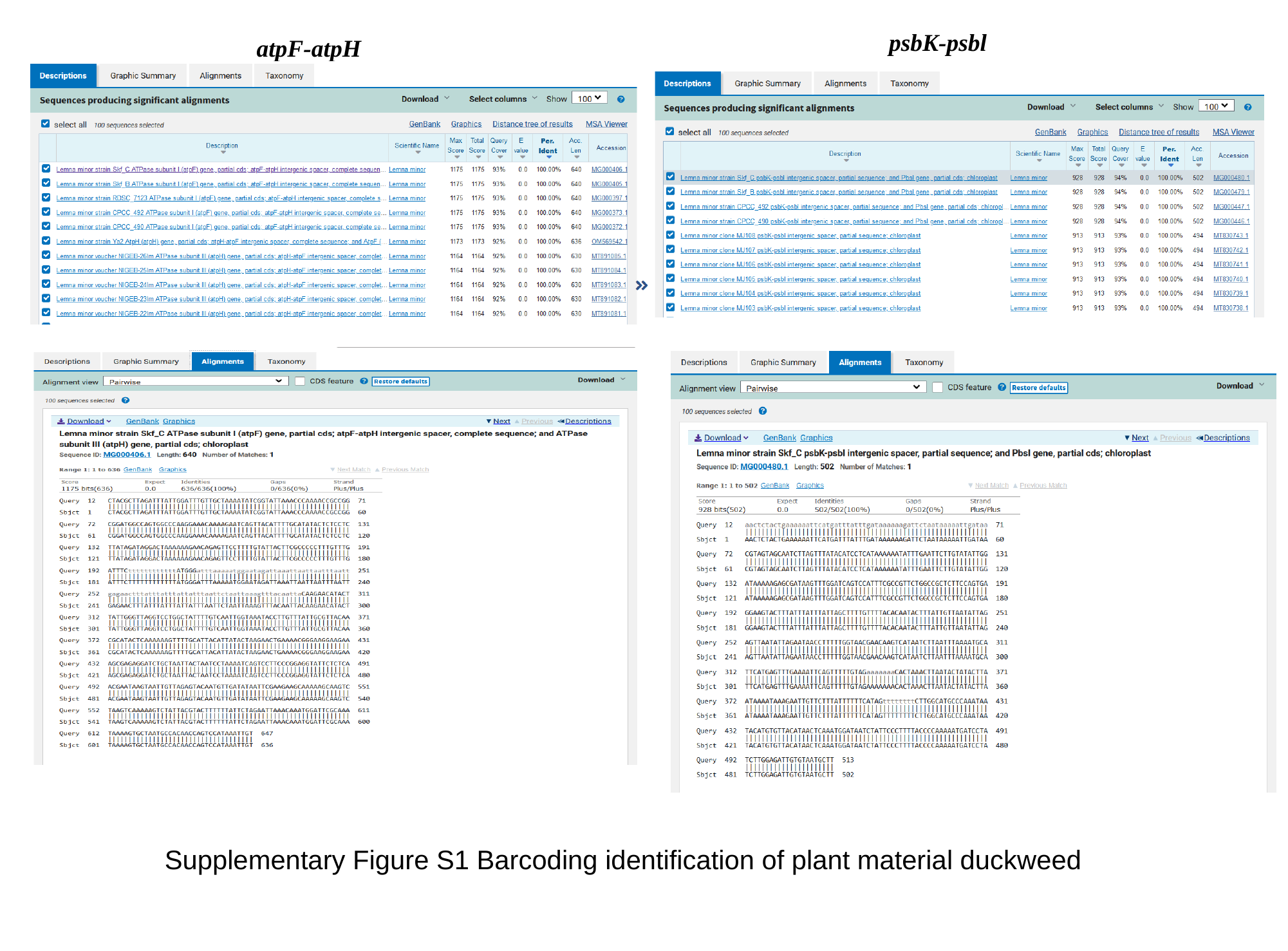

psbK-psbl
atpF-atpH
Supplementary Figure S1 Barcoding identification of plant material duckweed

## Slide 2
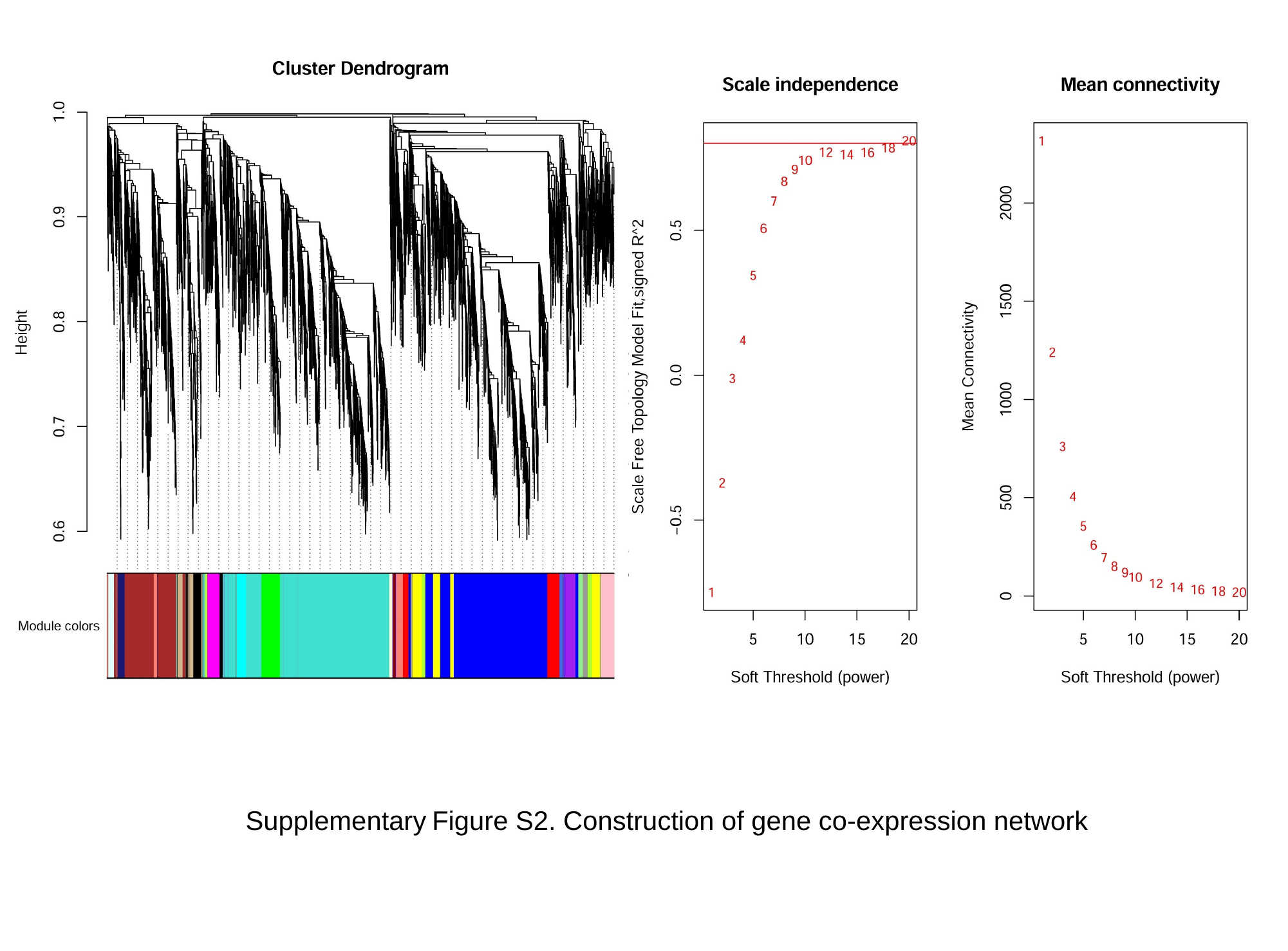

Supplementary Figure S2. Construction of gene co-expression network
